# Enhancing Neuroplasticity to Augment Cognitive Remediation in Schizophrenia

**DOI:** 10.3389/fpsyt.2017.00191

**Published:** 2017-09-27

**Authors:** Carol Jahshan, Yuri Rassovsky, Michael F. Green

**Affiliations:** ^1^VISN-22 Mental Illness Research, Education and Clinical Center (MIRECC), VA Greater Los Angeles Healthcare System, Los Angeles, CA, United States; ^2^Department of Psychiatry and Biobehavioral Sciences, David Geffen School of Medicine, University of California, Los Angeles, Los Angeles, CA, United States; ^3^Department of Psychology, Gonda Multidisciplinary Brain Research Center, Bar-Ilan University, Ramat-Gan, Israel

**Keywords:** schizophrenia, cognitive training, remediation, physical exercise, transcranial direct current stimulation, neuromodulation, neuroplasticity, brain-derived neurotrophic factor

## Abstract

There is a burgeoning need for innovative treatment strategies to improve the cognitive deficits in schizophrenia. Cognitive remediation (CR) is effective at the group level, but the variability in treatment response is large. Given that CR may depend on intact neuroplasticity to produce cognitive gains, it is reasonable to combine it with strategies that harness patients’ neuroplastic potential. In this review, we discuss two non-pharmacological approaches that can enhance neuroplasticity and possibly augment the effects of CR in schizophrenia: physical exercise and transcranial direct current stimulation (tDCS). Substantial body of evidence supports the beneficial effect of physical exercise on cognition, and a handful of studies in schizophrenia have shown that physical exercise in conjunction with CR has a larger impact on cognition than CR alone. Physical exercise is thought to stimulate neuroplasticity through the regulation of central growth factors, and current evidence points to brain-derived neurotrophic factor as the potential underlying mechanism through which physical exercise might enhance the effectiveness of CR. tDCS has emerged as a potential tool for cognitive enhancement and seems to affect the cellular mechanisms involved in long-term potentiation (LTP). A few reports have demonstrated the feasibility of integrating tDCS with CR in schizophrenia, but there are insufficient data to determine if this multimodal approach leads to incremental performance gain in patients. Larger randomized controlled trials are necessary to understand the mechanisms of the combined tDCS–CR intervention. Future research should take advantage of new developments in neuroplasticity paradigms to examine the effects of these interventions on LTP.

## Introduction

Antipsychotic medications are useful in ameliorating positive symptoms of schizophrenia, but they have little effect on cognitive deficits (1, 2). Efforts to improve cognitive deficits in schizophrenia are of paramount importance as they are among the strongest predictors of patients’ functional outcome (3). Most of the efforts for cognition enhancement have used pharmacological approaches (i.e., drugs that enhance learning and memory in animal models) (4). However, the results in larger trials have been disappointing, and currently, there is no drug approved for cognition enhancement in schizophrenia (5). Therefore, treatment studies in this population have started to shift to cognitive remediation (CR) strategies. Although CR in schizophrenia is effective at the group level (6, 7), there is substantial individual variability in treatment response, and many patients exhibit little benefit (8). Moreover, the training effects resulting from CR alone do not always generalize to improvements in real-life functioning (9). Thus, as the best validated treatment for the cognitive dysfunction in schizophrenia, CR only leads to a moderate effect-size improvement in cognition (0.45), with an even lower impact on daily functioning (0.36) (6, 9). It is, therefore, critical to consider ways of enhancing the impact of CR.

Recently, “neuroplasticity-based” interventions have been developed to train perceptual processes in schizophrenia, while also engaging attentional and working memory operations (10). These interventions are explicitly designed to drive adaptive plastic changes throughout distributed prefrontal–temporo–parietal systems (11). Many studies in schizophrenia patients have demonstrated that this neuroscience-informed approach to training generates meaningful restoration of prefrontal functions and higher-order cognition (12–14), with associated improvements in community functioning (15). Thus, neuroplasticity may be an important mechanism underlying effective intervention approaches. However, this CR method requires lengthy hours of repetitive, intensive practice to induce significant changes. Combining CR with strategies that promote neuroplasticity may not only lead to larger and longer-lasting improvements, but also require shorter training protocols. Although there have been efforts to combine CR with cognitive-enhancing medications that affect neuroplasticity, such as d-cycloserine (16) and modafinil (17), less attention has been devoted to non-pharmacological approaches that could potentially augment CR effects and maximize improvements in functional outcomes. In this brief review, we will discuss two recent non-pharmacological approaches that are thought to enhance neuroplasticity in schizophrenia: physical exercise and transcranial direct current stimulation (tDCS). It should be noted that there are many other neurostimulation techniques that have been discussed in the literature, such as transcranial magnetic stimulation (TMS), transcranial electrical stimulation (TES), magnetic seizure therapy, vagus nerve stimulation, and deep brain stimulation. However, only tDCS has been combined with CR.

## Review of Physical Exercise and tDCS Studies

### Physical Exercise and Cognition

The beneficial effects of physical exercise on cognition are well documented in healthy individuals, as well as across many medical and psychiatric illnesses (18–20). Accumulating evidence suggests that exercise reduces pro-inflammatory processes and peripheral risk factors (i.e., obesity and diabetes) that are associated with cognitive decline (21). Furthermore, it stimulates hippocampal neuroplasticity and promotes angiogenesis, neurogenesis, and synaptogenesis through the regulation of central growth factors (22). The mechanisms of exercise-induced cognitive improvements seem, to a large extent, to be related to an increased production of brain-derived neurotrophic factor (BDNF), which plays a pivotal role in synaptic plasticity and is particularly important for learning and memory (18, 23, 24). Similar to CR (25), exercise (26) has been shown to increase peripheral BDNF levels.

As the two approaches could potentially enhance cognition through overlapping neurobiological mechanisms, adding exercise to a CR program may further harness patients’ neuroplastic potential and lead to cognitive gains beyond that achieved by CR alone. The evidence from animal research suggests some benefit from combining these approaches. Fabel et al. (27), for example, showed that a combination of aerobic exercise and cognitive enrichment for rodents had beneficial effects on neurogenesis, leading to a 30% greater increase in new neurons than either activity alone. Several reports in healthy older adults have compared the separate vs. combined effects of CR and exercise and shown superior effects of the combined intervention on verbal/working memory (28, 29), divided attention (30), as well as global cognitive performance and everyday functioning (31). Studies in children (32) have also shown that training programs that integrate physical exercise with computer-based training games improved learning and increased gains on school-administered math and reading achievement tests.

### Physical Exercise in Schizophrenia

Physical activity has been shown to ameliorate the psychotic and negative symptoms of schizophrenia and improve patients’ quality of life by reducing health problems often associated with the illness (33, 34). Randomized controlled trials (RCTs) have been published recently demonstrating that physical exercise, especially aerobic exercise, improves cognitive functioning in schizophrenia patients, with corresponding increases in white matter integrity and structural connectivity (35), hippocampal volume (36), and BDNF signaling (37). A recent meta-analysis (38) identified 10 trials (7 RCTs and 3 non-randomized studies) evaluating the cognitive effects of exercise in schizophrenia. Pooled effect sizes across all outcomes showed that exercise significantly improved cognition (particularly attention, working memory, and social cognition) more than the control conditions. The treatment effect size of 0.33 (95% CI = 0.13–0.53, *p* = 0.001) across all studies and 0.43 (95% CI = 0.21–0.66, *p* < 0.001) in RCTs suggests that the beneficial effect of exercise on cognition in schizophrenia is comparable to that of CR.

### Physical Exercise plus CR in Schizophrenia

We are aware of three published studies that have combined CR and exercise in schizophrenia. In one study (39), 29 patients were randomly assigned to either CR and exercise or CR and mental relaxation. The interventions were 4-week long and consisted of three weekly sessions (30 min of CR and 45 min of either aerobic exercise or relaxation). Both groups showed cognitive gains in the domains of processing speed, working memory, and visual learning, improvement in subjective well-being, and reduction in negative symptoms. However, the effects were superior for the combined cognitive and physical training group.

In another study (40), 22 patients were enrolled in a 12-week endurance-training program augmented with CR and compared to a matched control group. Patients in the endurance training group exercised on bicycle ergometers, while those in the control group played table soccer for 30 min three times a week. After 6 weeks of the intervention period, CR was added in each group, for two 30 min-sessions a week. Results showed that, compared to the control condition, endurance training and CR significantly improved short- and long-term verbal memory, cognitive flexibility, global and social functioning, and negative symptoms. Unfortunately, the lack of random assignment, baseline differences between the groups, and the fact that the cognitive and clinical improvement in the combined training group was only seen after CR was added to the intervention make the findings difficult to interpret.

Last, a pilot study of recent-onset schizophrenia patients randomly assigned participants to 10 weeks of CR and exercise (*n* = 7) or CR alone (*n* = 9) (41). The CR intervention consisted of 2 h of auditory and social cognitive training twice/week, and exercise consisted of 30–45 min of aerobic conditioning 4 days/week. Results showed that the differential gains in global cognition and functional outcome were larger in the combined intervention group relative to the CR group, with Cohen’s *f* effect sizes of 0.48 for the MCCB overall composite and 0.88 for independent living skills.

### tDCS and Cognition

In recent years, neurostimulation has been developed as a non-invasive tool for cognitive enhancement (42, 43), with a primary advantage of having fewer side effects than pharmacological treatment (44). Unlike other brain stimulation techniques (e.g., TMS, TES), tDCS uses a weak electrical current (1–2 mA) to alter spontaneous neuronal network activity by shifting membrane potentials in a hyperpolarizing or depolarizing direction without inducing neuronal firing (45–47). tDCS changes the excitability of neurons in a polarity-dependent manner (48), such that anodal stimulation enhances cortical excitability, whereas cathodal stimulation decreases it (49). Various electrode montages can be applied to the scalp to modulate different areas of activity in the brain. Although tDCS has coarse spatial targeting, a few minutes of stimulation can lead to changes in cortical excitability lasting for over an hour (50).

The therapeutic effect of tDCS is thought to stem from its impact on the cellular and molecular mechanisms involved in long-term potentiation (LTP) (51, 52), and its after effects seem to be NMDA-receptor dependent (53). Thus, similar to physical exercise (21) and CR (54), tDCS appears to increase cortical plasticity (55) and could have additive or synergistic effects with CR, allowing for better cognitive outcomes.

A handful of studies in healthy samples have administered tDCS during specialized cognitive training and shown a performance-enhancing effect on the trained task (56) and generalization to untrained tasks (57). For instance, Martin et al. (56) demonstrated increased accuracy on a dual-working memory task during concurrent active tDCS vs. sham, but the effect was only present during the stimulation period and did not result in greater subsequent learning. This short-lived “online” effect has also been observed during combined tDCS and behavioral inhibition training (58). Additionally, Andrews et al. (57) found that completing an n-back task while receiving tDCS resulted in greater improvement in performance on Digit Span Forward compared to either tDCS or the cognitive activity alone. Similarly, the simultaneous administration of tDCS and computerized CR in healthy older adults significantly improved working memory compared with CR alone (59). The superiority in performance with this integrated approach was also evident in studies combining multiple repeated tDCS sessions with training on a motor skill task (52) and artificial numerical learning task (60).

### tDCS in Schizophrenia

Most studies examining the effects of tDCS in schizophrenia have administered the stimulation at rest, while the subject is engaged in a passive activity, such as watching a movie, followed by an “offline” assessment of interest. In different randomized sham-controlled trials, tDCS was found to enhance working memory (61), probabilistic association learning (62), and composite scores on measures of cognition (63), when applied to the left dorsolateral prefrontal cortex. More specifically, Hoy et al. (61) reported significantly better performance over time on a working memory task following a single tDCS session compared to sham (*p* = 0.027). Although Vercammen et al. (62) found no significant effect at the group level, a subgroup of patients with adequate learning at baseline improved with active tDCS. In Smith et al.’s RCT (63), active compared to sham tDCS subjects showed significant improvements after the fifth tDCS session in the MCCB overall composite (*p* = 0.008) and the working memory (*p* = 0.002) and attention-vigilance (*p* = 0.027) domain scores, with large effect sizes (Cohen’s *d* values ranged from 0.84 to 1.25). There is also evidence that tDCS can ameliorate auditory hallucinations (64, 65) and negative symptoms (66) in patients, as well as modulate the amplitude of the mismatch negativity, an EEG index of basic auditory processing (67).

### tDCS plus CR in Schizophrenia

Schizophrenia researchers have recently begun to investigate the feasibility and efficacy of integrating tDCS and CR. There are currently four published reports in this area, mostly piloting this procedure in small clinical samples. In one study (68), two patients received a neuroplasticity-based CR intervention combined with tDCS and showed cognitive improvements that were maintained at 1-month follow-up. The 4-week intervention consisted of five 45-min auditory training sessions a week with active tDCS administered concurrently with CR on three sessions per week. In another pilot study (69), patients (*n* = 10) received three working memory training sessions a week for 16 weeks, with active or sham tDCS applied during two of the CR sessions each week starting in week 3. The authors reported enhanced cognitive performance on word and picture N-back tasks and MCCB overall composite when CR was paired with tDCS. In a negative findings study (70), 10 patients were randomized to either active or sham tDCS (10 consecutive sessions over 5 days), with cognitive training (administration of n-back and sequence learning tasks) randomly applied during one of the tDCS sessions. The combined approach failed to improve clinical symptoms and cognitive performance.

In the largest study to date (71), investigators randomly assigned 49 patients to CR (training on a working memory and implicit learning task) and either active (*n* = 24) or sham tDCS (*n* = 25). The intervention was relatively short and consisted of four cognitive training days (day 1, day 2, day 14, and day 56), with two sessions on each day. tDCS was administered concomitantly with CR during the second session of days 1 and 14. Results showed significantly better working memory performance in the CR and active tDCS group relative to the CR and sham tDCS group. Surprisingly, the improved performance was evident on days 2 and 56, suggesting that tDCS had no enhancing effects during the acute stimulation but rather long-term effects on consolidation and learning.

## Conclusion and Future Directions

Based on the aforementioned review, it appears that both physical exercise and tDCS are intriguing candidates for augmenting the therapeutic effects of CR in schizophrenia. Current evidence suggests that a multimodal intervention that combines CR with physical exercise has a larger impact on cognitive functioning than CR alone. Moreover, there is strong evidence implicating BDNF as the mechanism underlying the cognitive-physical training approach (30, 41). Nonetheless, despite the promise that exercise has shown in augmenting CR in schizophrenia, there are several methodological issues that remain unresolved. For example, the literature is not consistent regarding the type, frequency, intensity, and duration of physical training necessary to produce the beneficial effects. Aerobic exercise has been the most studied and has produced the most consistent effects on cognition. However, other types of physical activity, such as yoga (72, 73), high-intensity interval training (74), and high-velocity circuit resistance training (75) deserve further attention. Furthermore, although Firth et al. (38) showed that a greater amount of exercise is associated with larger cognitive improvement, Kimhy et al. (76) found that it is the fidelity with target training intensity, rather than the frequency and duration of exercise, which correlates with changes in cognition. Some review studies (23, 72, 77) suggest a minimum of three sessions per week (at least 30 min/session) of moderate-intensity aerobic training for schizophrenia patients, administered in a supervised group setting for a minimum of 12 weeks, which is in line with recommendations by the American College of Sports Medicine (78).

In addition to refining optimal exercise training parameters, it is also essential to consider the timing of exercise with respect to CR when combining the two approaches. For instance, it might be more beneficial to start a treatment session with aerobic exercise followed by CR, as some studies have shown that engaging in physical activity before or while performing a cognitively demanding task improves learning or performance on the task (40). In a recent review (79), the authors proposed that aerobic exercise preceding CR may create a state of neuroplastic readiness in the brain through BDNF upregulation, which can potentiate the effectiveness of CR.

As far as the concurrent administration of tDCS and CR, emerging data support the feasibility and tolerability of this approach, but additional studies are needed to determine if it leads to performance gain in schizophrenia patients. Although the duration of stimulation of around 20 min has been consistently employed across studies, the therapeutic dose (i.e., number of sessions per day or week) has yet to be established. A host of parameters may moderate the effects of tDCS on cognitive outcomes, including placement and size of anodal/cathodal electrodes, unilateral vs. bilateral stimulation, amplitude of stimulation, and selection of training tasks during stimulation. Although it has been suggested that neuromodulation in combination with memory training may enhance the effects of training *via* LTP (80), the underlying mechanisms of tDCS have been mainly explored within the motor cortex and not memory-related regions. Therefore, beyond methodological research to identify a standard montage and the parameters required for therapeutic tDCS administration, larger RCTs are necessary to establish efficacy and relevant mechanisms of the combined tDCS–CR intervention.

Both approaches seem to have the potential to enhance the impact of CR by affecting functions that underlie neuroplasticity (55, 81). Fortunately, it is now possible to measure neuroplasticity *in vivo* in humans using neuroimaging techniques (e.g., EEG and fMRI). New paradigms have been recently developed to assess LTP non-invasively using repetitive sensory stimulation. Similar to electrical stimulation in animals (82), repetitive high-frequency stimulation (HFS) can induce LTP-like effects in humans (83–87). Some studies have measured LTP-like plasticity using a paradigm in which visual-evoked potentials (VEPs) to visual stimuli are recorded before and after the same stimulus is presented at a high frequency. Enhancement (increase in amplitude) of the VEPs after HFS is thought to reflect experience-dependent neuroplasticity of the visual cortex (84, 86–88). So far, two studies have been published using this EEG paradigm in schizophrenia (88, 89). Future treatment studies in schizophrenia should take advantage of these novel, non-invasive methods of assessing neuroplasticity to directly test whether physical exercise or tDCS affect LTP. For example, we are currently conducting an RCT in which a visual LTP paradigm is an outcome measure to examine changes in neuroplasticity following cognitive training.

In the absence of any robust pharmacological treatments for cognitive deficits in schizophrenia, physical exercise and tDCS are feasible and intriguing adjunctive treatments to enhance neuroplasticity and augment the effects of CR. While showing promise, their efficacy still needs to be demonstrated in more rigorously controlled studies.

## Author Contributions

CJ performed the literature search and drafted the manuscript. YR and MG critically reviewed the manuscript. All the authors read and approved the final manuscript.

## Conflict of Interest Statement

The authors declare that the research was conducted in the absence of any commercial or financial relationships that could be construed as a potential conflict of interest. The reviewers SI and ZN and handling editor declared their shared affiliation.
